# Health education *via* “empowerment” digital marketing of consumer products and services: Promoting therapeutic benefits of self-care for depression and chronic pain

**DOI:** 10.3389/fpubh.2022.949518

**Published:** 2023-01-10

**Authors:** Jade L. Huntsman, Grzegorz Bulaj

**Affiliations:** ^1^OMNI Self-care, LLC, Salt Lake City, UT, United States; ^2^L. S. Skaggs College of Pharmacy, University of Utah, Salt Lake City, UT, United States

**Keywords:** disease self-management, mental health, pain management, health promotion, health branding, health literacy, advertisement, digital media

## Abstract

Increasing health care costs and high economic burden exemplify the impact of chronic diseases on public health. Multifaceted approaches to treating chronic diseases include pharmaceutical drugs, digital therapeutics, and lifestyle medicine. Chronic diseases are largely preventable, and health promotion yields positive outcomes. However, despite positive return on investment (ROI) and cost-to-benefit ratio (CBR) for health promotion (median ROI 2.2, median CBR 14.4), commercial marketing of healthy lifestyles and self-care is limited. The objective of this perspective article is to discuss how digital marketing of consumer goods and services that support therapeutic self-care can also bridge public health and for-profit interests. We describe how “empowerment” marketing campaigns can provide evidence-based associations between products/services and self-care benefits for people living with chronic pain and depression. Such a “health education as marketing” strategy is illustrated by educational ads describing how contact with nature, music, and yoga can improve chronic pain and reduce depressive symptoms. Creating associations between health-related benefits of these activities with products (outdoor and yoga apparel, audio equipment) and services (music streaming services, music mobile apps, eco-tourism, yoga studios) that support them expand their value proposition, thus incentivizing profit-driven companies to engage in public health campaigns. Long-term success of companies that incorporate evidence-based health education as marketing and branding strategies will depend on following ethical considerations and advertising guidelines defined by consumer protection regulatory agencies, such as the Federal Trade Commission (FTC). In conclusion, integration of health education about self-care and commercial marketing can support health care outcomes and disease prevention.

## 1. Chronic diseases challenge public health and the economy

Six in ten people in the United States have a chronic medical condition, and four in 10 people have two or more (1). Chronic disease is the leading cause of disability and death in the U.S., contributing to the country's $3.8 trillion annual cost of healthcare (1). Chronic pain and depression are two chronic conditions impacting the health-related quality of life and productivity of millions of Americans each year. More than 100 million people live with chronic pain, affecting more people than cancer, diabetes, and heart disease combined (2). Depression is the leading cause of disability for ages between 15 and 44, affecting more than 16 million Americans 18 and older (3). The challenges of chronic diseases also include caregiver burden (4–7).

The economic costs of depression and chronic pain are estimated to be $210 billion (8) and between $560 and $635 billion, respectively, the latter alone surpassing the costs of heart disease, cancer, and diabetes (9). Costs include medical costs such as outpatient and inpatient medical services and pharmacy costs, and costs associated with loss of workplace productivity (8, 9). For example, 7.6% of the workforce is estimated to have depression and impaired job performance or chronic absenteeism as a result (8). Individuals experiencing moderate-to-severe chronic pain were found to work an average of 504 h fewer per year than individuals without pain (9).

A major challenge facing healthcare and caregivers today is meeting the complex needs of those living with chronic diseases (10). Dozens of surveys show that many people living with chronic diseases do not receive effective therapy, have poor disease control, and are dissatisfied with their care (10). Treatment of chronic diseases, including chronic pain and depression, largely relies on pharmacotherapies, while recent emergence of digital health technologies, including digital therapeutics, offers additional means to improve therapy outcomes (11–14). However, current efforts to treat and prevent chronic disease in the U.S. lack adequate resources. A 2005 assessment on chronic disease programs in the U.S. found that although chronic disease accounts for 70% of deaths nationwide, health departments allocate an average of 1.85% of their budget to chronic diseases (15). The Center for Disease Control and Prevention (CDC), one of the primary federal resources for funding public health activities in the U.S., has <$3 per capita to spend on chronic disease prevention (16). The prevention and public health fund was allotted <10% of the total CDC funding for programs in 2020, and this number has remained steady over time, despite increasing evidence for prevention program efficacy (16).

The objective of this perspective article is to describe how promoting therapeutic benefits of self-care *via* digital marketing of consumer goods and services can also bridge public health and for-profit interests. In the following sections, we discuss: (1) why chronic diseases are preventable, (2) how health education and promotion can impact health-related behaviors, (3) therapeutic benefits of self-care, (4) examples of digital marketing to promote self-care related to depression and chronic pain, and (5) opportunities and limitations of a “health education as marketing” approach. We hope that this cross-disciplinary work will advance future theoretical frameworks and translational studies on promotion of self-care practices. We also hope that this paper will encourage private entities and public-private partnerships to expand education about health benefits of self-care.

## 2. Chronic diseases and related mortality are largely preventable

Preventable chronic diseases prematurely kill more than 1.7 million Americans each year (17–19). Chronic diseases largely result from modifiable lifestyle choices including tobacco use, poor diet, lack of physical activity, excessive alcohol consumption, and uncontrolled high blood pressure (17). The World Health Organization (WHO) predicts that if the major preventable causes of chronic diseases were eliminated, 80% of heart disease, stroke, and type 2 diabetes, and more than 40% of cancer cases would be prevented (20). Ineffective management of chronic pain and depression render two examples of how chronic disease-related deaths are largely preventable. Currently, the most common form of pain relief in the U.S. is opioid prescriptions, and over 49 million Americans filled at least one opioid prescription in 2018 (21). The National Institute on Drug Abuse reported that 21–29% of patients prescribed opioids for chronic pain management misuse them, contributing to approximately 50,000 opioid overdose deaths in 2019 (22). Similarly, evidence shows that depression is the leading cause of avoidable suffering worldwide (23), with a 33% increase in suicide rates between 1999 and 2019, making suicide the 10th leading cause of death in the U.S., accounting for over 47,500 deaths in 2019 (24). An analysis of healthcare outcomes comparing the U.S. to 10 other high-income countries found that the U.S. not only yields the lowest performance despite spending a significantly larger percentage of GDP on healthcare, but also ranks last on preventable mortality with only a 5% mortality reduction compared to all other countries averaging a 20% reduction over a recent period of 10 years (25).

## 3. Health promotion, education, and empowerment

In this perspective article, we describe how bridging digital marketing with health promotion, health education, and empowerment can positively impact public health and disease prevention. Health promotion is defined by WHO as “*The process of enabling people to increase control over, and to improve their health*”, and health education is defined as “*consciously constructed opportunities for learning…designed to improve health literacy, including improving knowledge, and developing life skills which are conducive to individual and community health*” (26). Empowerment is defined as “*a process through which people gain greater control over decisions and actions affecting their health*” (26). All three, health promotion, education, and empowerment aim to foster healthy lifestyles, as well as motivation and confidence necessary to take action to improve one's health.

Research has shown the effectiveness of health promotion, education, and empowerment on increasing health-related behaviors and overall health outcomes (27–30). A single two-hour educational empowerment session, facilitated *via* Zoom, had substantial and long-lasting positive effects on pain reduction and management for individuals living with chronic pain (31, 32). A program called Creating Opportunities for Personal Empowerment was effective in reducing depression and anxiety and increasing academic performance among college students (33). A growing body of research shows positive outcomes of school-based mental health promotion programs in areas such as mental health literacy and resilience enhancement (34, 35). Studies on workplace health promotion provide evidence that health promotion can achieve long-term behavior change and risk reduction (36–38).

Social media is increasingly being used for health promotion (39–41), and digital marketing can influence health-related behaviors and beliefs (42–45). A randomized controlled study of a Facebook-based campaign targeting mental health literacy and help-seeking attitude for depression reported positive findings (46). Social media campaigns promoting mental health awareness gained more recognition during the COVID-19 pandemic (47). Media campaigns promoting chronic pain management are promising but underexplored tools toward changing related beliefs and behaviors (48). A number of internet-delivered programs for disease prevention and treatment, including mental health and chronic pain, are available as low-cost interventions and educational resources (49).

While public health benefits of health promotion and education are apparent, cost-benefit analysis of health promotion also shows positive outcomes (30, 50–53). A systematic review of the return on investment (ROI) and cost-to-benefit ratio (CBR) for public health interventions indicates that ROI for health promotion ranged from 0.6 to 6.2 (median ROI was 2.2 based on 12 studies) and CBR ranged from 2 to 29.4 (median CBR 14.4 based on 3 studies) (54) [“*The CBR is the benefit divided by the cost, and the ROI is the benefit minus the cost expressed as a proportion of the cost*…” (54)]. While economic evaluation of mental health promotion programs shows promising results, their cost-effectiveness remains inconclusive due to limited number of relevant studies (55). In this perspective article, our main objective is to bridge health promotion with commercial interests by describing digital marketing of consumer goods and services associated with self-care.

## 4. Lifestyle medicine and therapeutic benefits of self-care

Lifestyle medicine involves behavioral, environmental, and motivational factors to improve physical and mental health (56, 57). Lifestyle interventions and self-care have been gaining recognition for their abilities to produce clinical effects in people living with chronic medical conditions. In 2017, the American College of Physicians published clinical guidelines recommending yoga and physical exercise as the first-line therapy for chronic low back pain (58). An increasing number of studies support health benefits of diverse self-care practices ranging from contact with nature (e.g., forest bathing, walking/hiking outside), yoga practice, mindfulness meditation, breathing exercises, listening to music, aromatherapy, sleep hygiene, and others. Lifestyle interventions have been reported for depression and anxiety (57, 59, 60), chronic pain (61, 62), arthritis (63), cancer (59), dementia and Alzheimer's disease (64), and diabetes (65, 66).

Using PubMed and Google Scholar databases, we searched for reviews and clinical research studies that investigated therapeutic benefits of selected self-care practices for pain and depression. As summarized in Table 1, contact with nature, yoga practice, and music-based interventions have been shown to reduce chronic pain and improve depressive symptoms. Research studies show health-related benefits of exposure to nature (67), including improvements in cognitive and mental health (68–72), chronic pain conditions (73), and immune and cardiovascular functions (74, 75). Similarly, listening to music and yoga practice exert multiple physiological and clinical effects ranging from pain relief and pain management to reducing depressive symptoms and modulating the immune system (76–85). As we discuss below, therapeutic benefits of self-care practices can be disseminated *via* digital marketing and creating knowledge-based associations between specific self-care practices and products or services that support these practices.

**Table 1:** Examples of self-care practices and related products and services that can benefit people living with chronic pain and depression.

**Self-care practices**	**Examples of therapeutic benefits of self-care**	**Examples of products/services supporting self-care (companies offering related products/services)**
Contact with nature	Two SR/MA studies suggested that nature-based interventions, including forest bathing, can reduce depressive symptoms and improve positive affect (68, 86). Diverse nature interventions reduced pain and fatigue in people with fibromyalgia (87), while forest bathing can significantly reduce chronic neck pain (73).	Outdoor apparel and equipment supporting contact with nature (Arc'teryx, Backcountry, Black Diamond, Columbia Sportswear, Cotopaxi, L.L. Bean, Mountain Hardwear, Naturalist Ventures, Outdoor Research, Patagonia, REI, Salomon, The North Face)
Yoga	SR/MA suggested that yoga practice can reduce depressive symptoms among people with cancer (80). SR/MA showed yoga practice can reduce prenatal depression (88). SR/MA studies showed yoga practice can reduce chronic low back pain and disability (81), and can also reduce pain intensity and frequency/duration of migraine headaches (89). Clinical practice guidelines published by the American College of Physicians recommend yoga as first line therapy for chronic low back pain (58).	Yoga apparel, studios, and retreats supporting yoga practice (Athleta, CorePower, Gaiam, Honor Yoga, Life Time, Lululemon, Real Hot Yoga, Sunstone Yoga, The Travel Yogi, Truenature Travels, YogaFit, Yogascapes, YogaSix, Yoga to the People, YogaWorks)
Music	Systematic review of diverse music interventions revealed significant reduction in depression symptoms (76). Meta-analysis showed diverse music therapy and music medicine interventions can significantly reduce depressive symptoms (77). SR/MA showed music-based interventions can produce analgesic effects in chronic pain conditions (78). Meta-analysis showed music interventions can produce pain relief and decreased use of analgesic medications (79).	Music streaming services and mobile apps delivering music, and audio equipment (Amazon Music, Apple Music, Bose, Deezer, Harman Kardon, JBL MixCloud, Primephonic, Qobuz, Sony, SoundCloud, Spotify, Tidal, YouTube Music)

Examples of therapeutic benefits of self-care are based on systematic reviews and meta-analyses (SR/MA), other review and clinical research studies. Selected examples of companies associated with the products and services that can provide direct or indirect therapeutic benefits are also listed. Please note that the names of the companies listed are under trademark and copyright protection.

## 5. Digital marketing of consumer products and services promoting health benefits of self-care

Commercial marketing of healthy lifestyles by businesses and public-private partnerships has been primarily focused on obesity, nutrition, and physical activity (90). Herein, we describe a “health education as marketing” strategy to promote consumer goods and services associated with diverse self-care practices. We provide examples of how for-profit businesses can expand the scope of marketing campaigns by educating customers about connections between their products/services and health-benefits of self-care. Using two examples of chronic diseases, namely chronic pain and depression, and three examples of self-care practices, namely contact with nature, yoga practice, and listening to music, we describe associations between products/services supporting the aforementioned practices and their clinical benefits. We discuss such “empowerment” marketing campaigns as public health interventions delivering evidence-based information about health benefits of self-care.

As shown in Table 1, contact with nature (forest bathing, spending time in green and blue spaces), listening to music, and yoga practice provide clinical benefits related to chronic pain management and reduction of depressive symptoms. Each of these three activities is associated with specific products and services that support daily self-care to reduce chronic pain or depression. For example, camping in the forest, hiking in the mountains, enjoying water activities at the lake, and eco-tourism are enabled by proper apparel and equipment. Similarly, participation in yoga practice includes the use of yoga apparel and equipment, as well as attending yoga classes and retreats. Listening to music is facilitated by a combination of music streaming services, mobile apps, smartphones, and audio equipment including headphones, earphones, and audio speakers. Current marketing and advertising campaigns of the forementioned products/services are commonly focused on brand awareness and/or enjoyment of specific products and services. For example, outdoor apparel and equipment companies emphasize that contact with nature is much easier and more satisfying with the proper gear (hence people are more likely to do it).

**Figure 1:**
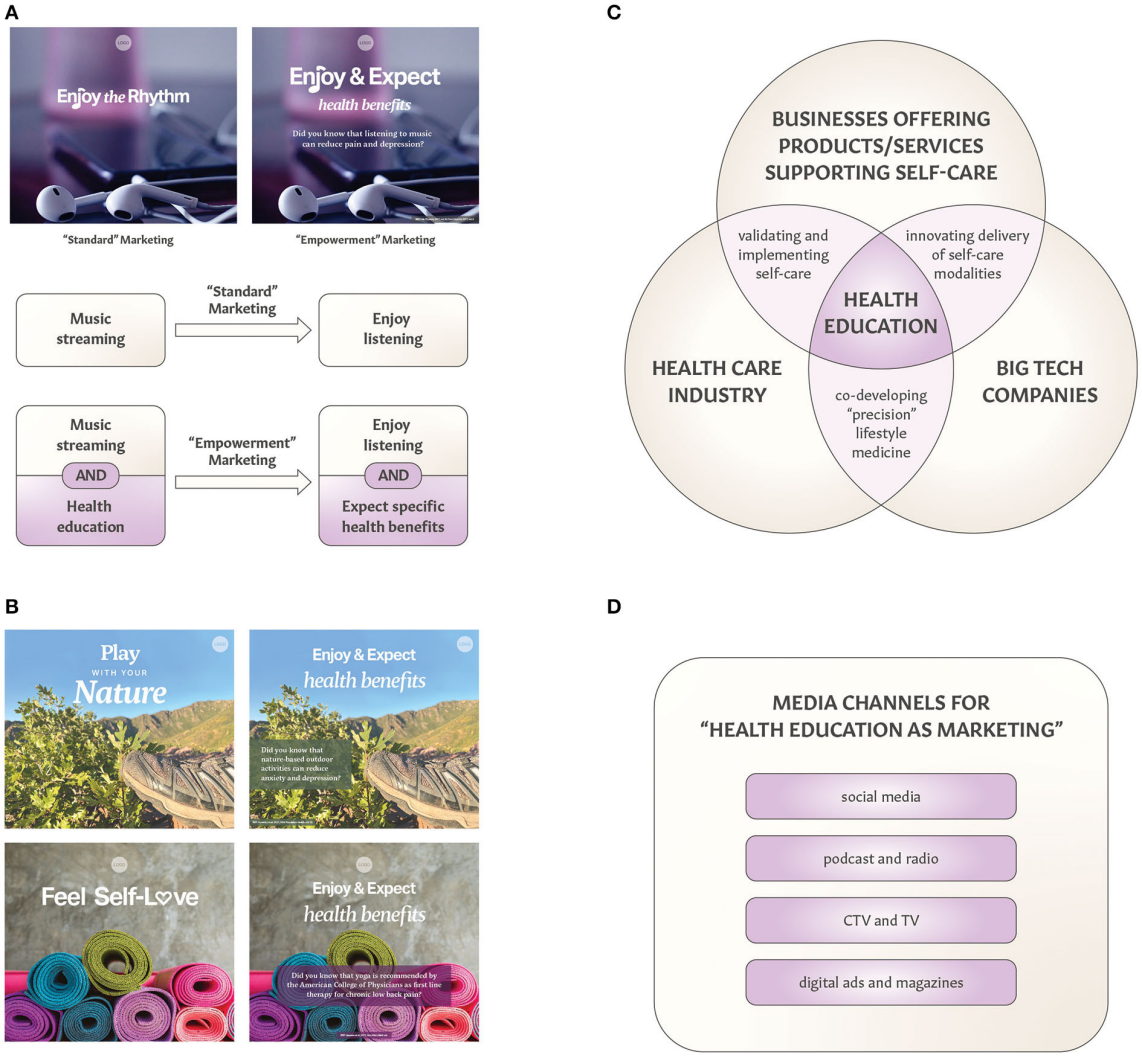
“Health education as marketing” for consumer products and services. **(A)** Comparing “standard” marketing with health-focused “empowerment” marketing of music streaming (or audio equipment) illustrates opportunities to build associations between listening to music and receiving such health benefits as reduction of pain or depression. The statement shown in this digital ad “Did you know that listening to music can reduce pain and depression” is supported by systematic review and meta-analysis studies referenced in Table 1. **(B)** Examples of “standard” and “empowerment” marketing of products and services associated with contact with nature and yoga practice. The statements shown in these digital ads “Did you know that nature-based outdoor activities can reduce anxiety and depression?” and “Did you know that yoga is recommended by the American College of Physicians as first line therapy for low back pain?” are supported by references listed in Table 1. **(C)** Collaborative opportunities for three key stakeholders to develop, validate, implement, and scale-up “health education as marketing” strategies. Alliances between commercial companies and health care can include industry-sponsored clinical studies supporting personalized (“precision”) lifestyle interventions, whereas big tech companies can contribute to innovating products and services supporting self-care. To bridge commercial interests with public health, promoting benefits of self-care can be a part of marketing and branding strategies. Engaging multiple stakeholders in co-creating health promotion was recently described (91). **(D)** Diversity of digital media channels applicable for “health education as marketing” to promote self-care benefits. CTV stands for connected TV.

Table 1 lists examples of companies that offer apparel and equipment enabling consumers to spend time in nature, practice yoga, or listen to music. While marketing their products and services, these companies have an opportunity to bridge their marketing and advertising with public education about health benefits of relevant self-care practices. Figures 1A, B illustrate the difference between “standard” marketing and “empowerment” marketing of consumer products/services associated with exposure to nature, yoga practice, or listening to music. The “standard” marketing of music-related products and services is focused on engagement with and enjoyment of music, whereas the “empowerment” marketing is focused on educating consumers about health benefits of music-related products/services. It is noteworthy that the general public appreciates the ability of music to elevate mood, but there is less awareness of the analgesic and antidepressant properties of music (references in Table 1).

Such health-focused “empowerment” marketing and health branding approach can also be applied to products and services that facilitate contact with nature and yoga practice (Figure 1B). The general public is largely unaware that yoga and physical exercise are recommended by the American College of Physicians as the first line therapy for chronic low back pain (58). Such a gap in public knowledge creates an opportunity for yoga studios and companies manufacturing and promoting yoga apparel to create “empowerment” marketing and digital ads that offer relevant and evidence-based information (Figure 1B). Since nature therapies such as eco-therapy and forest bathing gain more recognition for their clinical effects (92, 93), advertising health benefits of connection with nature can be a part of health promotion campaigns (94). It is noteworthy that “positive-gain” message framing has been shown to be more effective in promoting health-related behaviors (95, 96). As discussed below, creating associations between health-related benefits and products (outdoor equipment, yoga products and apparel, audio equipment) and services (music streaming, eco-tourism, yoga retreats) can also expand their value proposition.

From a practical perspective, companies offering outdoor apparel and equipment can expand their “standard” advertising strategy (e.g., The North Face^®^ “Never Stop Exploring^®^” and “Get Outdoors” marketing campaigns) by adding information about mental health benefits of spending time in the forest. The “standard” marketing for music streaming companies (e.g., ads bridging music and real-life with fun memes and the “Listening is everything” campaign by Spotify) could be expanded by providing “real-life” information about clinical studies indicating that listening to music can reduce depression or pain. Another example is Lululemon which presents itself as “a global company rooted in yoga” that aims “to contribute to conditions that support physical, mental, and social wellbeing” through their “Be Well” Impact Agenda (quotes from corporate.lululemon.com). Lululemon campaigns such as “Feel” or “This is Yoga” focus on wellbeing and “Practice of self-discipline” or “Practice of breath”. As illustrated in Figure 1B, marketing agencies can consider “empowerment” advertising of yoga apparel and activewear by providing information about yoga being recommended by the American College of Physicians as the first line therapy for chronic low back pain since 2017 (58).

## 6. Opportunities and limitations of “health education as marketing”

The “health education as marketing” strategy offers new opportunities for commercial businesses such as cross-disciplinary collaborations with the health care industry (Figure 1C), while expanding value proposition of products and services supporting self-care. Collaborations between commercial businesses and healthcare systems can focus on clinical studies validating therapeutic outcomes of specific self-care practices, for example, testing efficacy and effectiveness of music streaming on depression (97, 98). Joint-venture alliances between music streaming services, digital health companies, and clinical research centers could lead to expanding value proposition for music because of its additional therapeutic applications (97–100). In addition to the antidepressant and analgesic properties of music, the ability of musical compositions to reduce seizures in people with epilepsy can also be translated to clinical practice after pivotal RCTs and the regulatory approval by the FDA (101, 102). Industry-sponsored clinical studies, including large-scale RCTs, will contribute toward high-quality, rigorous evidence of self-care benefits for specific chronic diseases, ultimately contributing to lifestyle medicine and better therapy outcomes.

Currently health-targeted marketing is a multi-billion dollar industry in the U.S., led by direct-to-consumer advertising of prescription drugs and health services (103). The “health education as marketing” strategy can increase value proposition for products and services supporting such self-care practices as contact with nature and yoga, since consumers who consider purchasing related products/services would more likely choose those that also provide information about the health benefits of using them (104, 105). Promoting “contact with nature” has already been recognized as an important public health intervention to prevent mental illness (94), therefore, marketing outdoor apparel and equipment in the context of improving mental health can also increase its value proposition. Similarly, yoga studios and companies producing yoga apparel have opportunities to create associations between their products/services and respective health benefits (Table 1). One example of expanding value proposition of otherwise commercial services is biophilic interior design fostering self-care for people living with migraines, chronic pain, and depression (106).

Digital marketing includes diverse types and strategies (107). Figure 1D illustrates examples of digital media channels applicable for “health education as marketing” campaigns. In addition to more traditional channels (e.g., social media ads, digital ads, and radio/TV ads), there are expanded opportunities for promoting health benefits of self-care *via* connected TV (CTV) ads, digital magazines, and podcasts. Video- and audio-based channels can be used for digital storytelling, which has already been used as education, marketing, empowerment, and health promotion tools (108–111). It is noteworthy that multimedia communications enable more effective outreach covering diverse demographics and interest groups.

There are apparent limitations to developing and implementing “health education as marketing” campaigns, which include ethical, regulatory, and methodological aspects (103, 112–117). All three aspects are interconnected, while ethical and regulatory aspects are also related to the quality of evidence based on which health-related claims are communicated as marketing messages. When delivering health-related information in commercial ads, it is critical to avoid deceptive marketing practices leading to consumers' false beliefs and eventually impacting a company's value (118). It is important that the information presented is truthful and substantiated, as stated by the Federal Trade Commission (FTC), while claims must be backed by sound, unbiased and the state-of-the-art scientific evidence. The FTC monitors for violations of fair advertising practices. Ethical issues of using digital media in public health and greater consumer protection from deceptive digital marketing have been acknowledged (119, 120). While self-care practices discussed here are considered relatively safe, it is important to emphasize that any misinterpretation of health promotion delivered as marketing information can place some individuals at risk. Implementing public health risk/benefit assessment will help to inform all stakeholders about long-term outcomes of “health education as marketing” campaigns.

The balance between opportunities and limitations of the “health education as marketing” strategy will define development, implementation, and scale-up of digital marketing and advertising campaigns for diverse consumer products and services. Herein, we showed only examples of products/services associated with nature, yoga and music. Additional prospects include products and services associated with physical exercise (wellness and fitness industry), aromatherapy, sleep hygiene, and diverse forms of relaxation as means to reduce stress.

## 7. Conclusions

In this perspective article, we describe opportunities to expand health education and promotion related to self-care. Public health and non-profit spending is inadequate to bridge the gap between the prevalence of chronic diseases and positive outcomes of health promotion, thus necessitating more engagement from the commercial sector (e.g., private and public companies and corporations). The promotion of health benefits of self-care can be incorporated into commercial marketing of products and services related to contact with nature, yoga practice, and listening to music. These self-care modalities have been shown to reduce chronic pain and depressive symptoms, leading to better treatments and prevention of these two major chronic diseases. The development and implementation of the “health promotion as marketing” strategy requires integration of rigorous scientific evidence and ethical marketing strategies. We hope that this article will contribute to advancing public health interventions aiming to reduce chronic disease prevalence and burden.

## Data availability statement

The original contributions presented in the study are included in the article, further inquiries can be directed to the corresponding author.

## Author contributions

GB conceived the project. JH and GB performed the literature review and wrote the manuscript. Both authors contributed to the article and approved the submitted version.

## References

[1] Chronic Diseases in America. (2021). Available online at: https://www.cdc.gov/chronicdisease/resources/infographic/chronic-diseases.htm (accessed September 20, 2021).

[2] The Silver Book: Persistent Pain. Chronic Disease and Medical Innovation in an Aging Nation. Washington, DC: The Silver Book (2013).

[3] Anxiety Depression Association of America: Facts. Anxiety and Depression Association of America. (2021). Available online at: https://www.adaa.org (accessed December 19, 2022).

[4] Adelman RD Tmanova LL Delgado D Dion S Lachs MS. Caregiver burden: a clinical review. JAMA. (2014) 311:1052–60. 10.1001/jama.2014.304 24618967

[5] Donohue JM Pincus HA. Reducing the societal burden of depression: a review of economic costs, quality of care and effects of treatment. Pharmacoeconomics. (2007) 25:7–24. 10.2165/00019053-200725010-00003 17192115

[6] Zivin K Wharton T Rostant O. The economic, public health, and caregiver burden of late-life depression. Psychiatr Clin North Am. (2013) 36:631–49. 10.1016/j.psc.2013.08.008 24229661 PMC4024243

[7] Balkaran BL. Self-reported burden of caregiver of adults with depression: a cross-sectional study in five Western European countries. BMC Psychiatry. (2021) 21:312. 10.1186/s12888-021-03255-6 34154555 PMC8215758

[8] Greenberg PE Fournier A-A Sisitsky T Pike CT Kessler RC. The economic burden of adults with major depressive disorder in the United States (2005 and 2010). J Clin Psychiatry. (2015) 76:155–62. 10.4088/JCP.14m09298 25742202

[9] Gaskin DJ Richard P. The economic costs of pain in the United States. J Pain. (2012) 13:715–24. 10.1016/j.jpain.2012.03.009 22607834

[10] Wagner EH. Chronic disease management: what will it take to improve care for chronic illness? Effective Clinical Practice. (1998) 1:2–4. 10345255

[11] Sverdlov O van Dam J Hannesdottir K Thornton-Wells T. Digital therapeutics: an integral component of digital innovation in drug development. Clin Pharmacol Ther. (2018) 104:72–80. 10.1002/cpt.1036 29377057

[12] Patel NA Butte A Characteristics J and and challenges of the clinical pipeline of digital therapeutics. NPJ Digit Med. (2020) 3:159. 10.1038/s41746-020-00370-8 33311567 PMC7733514

[13] Velez FF Malone DC. Cost-effectiveness analysis of a prescription digital therapeutic for the treatment of opioid use disorder. J Mark Access Health Policy. (2021) 9:1966187. 10.1080/20016689.2021.1966187 34434535 PMC8381930

[14] Rubin R. Virtual reality device is authorized to relieve back pain. JAMA. (2021) 326:2354–2354. 10.1001/jama.2021.22223 34932094

[15] Georgeson M Thrope L Merlino M Freidman T Fieldnig J. Shortchanged? An assessment of chronic disease programming in major US city health departments. J Urban Health. (2005) 82:183–90. 10.1093/jurban/jti042 15890761 PMC3456563

[16] The Impact of Chronic Underfunding on America's Public Health System: Trends Risks, Recommendations, 2020. Trust for America's Health. (2020). Available online at: https://tfah.org (accessed December 19, 2022).

[17] Bauer UE Briss PA Goodman RA Bowman BA. Prevention of chronic disease in the 21st century: elimination of the leading preventable causes of premature death and disability in the USA. Lancet. (2014) 384:45–52. 10.1016/S0140-6736(14)60648-6 24996589

[18] Fraser. Leading chronic disease prevention with evidence-based policy: the ASTHO example. J Public Health Manag Pract. (2020) 26:506–9. 10.1097/PHH.0000000000001242 32732727

[19] Tinker A. How to improve patient outcomes for chronic diseases and comorbidities. Health Catalyst. (2017). Available online at: https://healthcatalyst.com (accessed December 19, 2022).

[20] World Health Organization. Preventing chronic diseases: a vital investment: WHO global Report. Geneve, Switzerland: WHO Press. (2005).

[21] Centers for Disease Control Prevention. (2019). Annual Surveillance Report of Drug-Related Risks and Outcomes–United States Surveillance Special Report. Centers for Disease Control and Prevention, U.S. Department of Health and Human Services. Available online at: https://www.cdc.gov/drugoverdose/pdf/pubs/2019-cdc-drug-surveillancereport.pdf (accessed December 18, 2022).

[22] Opioid Overdose Crisis. (2021). Available online at: https://www.drugabuse.gov/drug-topics/opioids/opioid-overdose-crisis (accessed March 11, 2021).

[23] Herrman H Patel V Kieling C Berk M Buchweitz C Cuijpers P. Time for united action on depression: a Lancet–World Psychiatric Association Commission. The Lancet. (2022) 399:957–1022. 10.1016/S0140-6736(21)02141-3 35180424

[24] Preventing Suicide. Centers for Disease Control and Prevention, National Center for Injury Prevention Control. (2021). Available online at: https://cdc.org (accessed December 19, 2022).

[25] Young JD Bhashyam AR Qudsi RA Parisien RL Shrestha S van der Vliet QMJ. Reflecting Poorly: Health Care in the US Compared to Other High-Income Countries. New York, NY: The Commonwealth Fund. (2021).

[26] Nutbeam D. Health promotion glossary. Health Promot Int. (1998) 13:349–64. 10.1093/heapro/13.4.349

[27] Proper KI van Oostrom SH. The effectiveness of workplace health promotion interventions on physical and mental health outcomes - a systematic review of reviews. Scand J Work Environ Health. (2019) 45:546–59. 10.5271/sjweh.3833 31134284

[28] Lee M Lee H Kim Y Kim J Cho M Jang J. Mobile app-based health promotion programs: a systematic review of the literature. Int J Environ Res Public Health. (2018) 15:2838. 10.3390/ijerph15122838 30551555 PMC6313530

[29] Rudolf K Dejonghe LAL Froböse I Lammer F Rückel L-M Tetz J. Effectiveness studies in health promotion: a review of the methodological quality of studies reporting significant effects on physical activity in working age adults. Int J Environ Res Public Health. (2019) 16:813. 10.3390/ijerph16050813 30845673 PMC6427597

[30] MacMonegle AJ Nonnemaker J Duke J Farrelly M Zhao X Smith A. Cost-effectiveness analysis of the real cost campaign's effect on smoking prevention. Am J Prev Med. (2018) 55:319–25. 10.1016/j.amepre.2018.05.006 30122214 PMC13374720

[31] Ziadni MS Gonzalez-Castro L Anderson S Krishnamurthy P Darnall BD. Efficacy of a single-session “empowered relief” zoom-delivered group intervention for chronic pain: randomized controlled trial conducted during the COVID-19 pandemic. J Med Internet Res. (2021) 23:e29672. 10.2196/29672 34505832 PMC8463950

[32] Darnall BD Roy A Chen AL Ziadni MS Keane RT You DS. Comparison of a Single-Session Pain Management Skills Intervention With a Single-Session Health Education Intervention and 8 Sessions of Cognitive Behavioral Therapy in Adults With Chronic Low Back Pain: A Randomized Clinical Trial. JAMA Netw Open. (2021). 4:e2113401. 10.1001/jamanetworkopen.2021.13401 34398206 PMC8369357

[33] Abney BGH Lusk P Hovermale R Melnyk BM. Decreasing depression and anxiety in college youth using the creating opportunities for personal empowerment program (COPE). J Am Psychiatr Nurses Assoc. (2019) 25:89–98. 10.1177/1078390318779205 29865903

[34] Dahlberg. Systematic review of resilience-enhancing, universal, primary school-based mental health promotion programs. BMC Psychol. (2018) 6:30. 10.1186/s40359-018-0242-3 29976252 PMC6034212

[35] Campos L Dias P Duarte A Veiga E Dias CC Palha F. Is it possible to “find space for mental health” in young people? effectiveness of a school-based mental health literacy promotion program. Int J Environ Res Public Health. (2018) 15:1426. 10.3390/ijerph15071426 29986444 PMC6069495

[36] Goetzel RZ Ozminkowski RJ. The health and cost benefits of work site health-promotion programs. Annu Rev Public Health. (2008) 29:303–23. 10.1146/annurev.publhealth.29.020907.090930 18173386

[37] Basińska-Zych A Springer Organizational A, and individual outcomes of health promotion strategies-a review of empirical research. Int J Environ Res Public Health. (2021) 18:383. 10.3390/ijerph18020383 33419033 PMC7825322

[38] Pham CT Phung D Nguyen TV Chu C. The effectiveness of workplace health promotion in low- and middle-income countries. Health Promot Int. (2020) 35:1220–9. 10.1093/heapro/daz091 31495871

[39] O'Reilly M Dogra N Hughes J Reilly P George R Whiteman N. Potential of social media in promoting mental health in adolescents. Health Promot Int. (2019) 34:981–91. 10.1093/heapro/day056 30060043 PMC6904320

[40] Moorhead SA Hazlett D Harrison L Carroll JK Lrwin A Hoving C. A new dimension of health care: systematic review of the uses, benefits, and limitations of social media for health communication. J Med Internet Res. (2013) 15:e85. 10.2196/jmir.1933 23615206 PMC3636326

[41] Günther L Schleberger S Pischke CR. Effectiveness of social media-based interventions for the promotion of physical activity: scoping review. Int J Environ Res Public Health. (2021) 18:24. 10.3390/ijerph182413018 34948628 PMC8702047

[42] Buchanan L Kelly B Yeatman H Kariippanon K. The effects of digital marketing of unhealthy commodities on young people: a systematic review. Nutrients. (2018) 10:148. 10.3390/nu10020148 29382140 PMC5852724

[43] Scully M Dixon H Wakefield M. Association between commercial television exposure and fast-food consumption among adults. Public Health Nutr. (2009) 12:105–10. 10.1017/S1368980008002012 18339226

[44] De Veirman M Hudders L Nelson MR. What is influencer marketing and how does it target children? A review and direction for future research. Front Psychology. (2019) 10:2685. 10.3389/fpsyg.2019.02685 31849783 PMC6901676

[45] Murphy G Corcoran C Golden M Boyland E Rooney B. See, like, share, remember: adolescents' responses to unhealthy-, healthy- and non-food advertising in social media. Int J Environ Res Public Health. (2020) 17:7. 10.3390/ijerph17072181 32218252 PMC7177346

[46] Wong. Evaluation of an online campaign for promoting help-seeking attitudes for depression using a facebook advertisement: an online randomized controlled experiment. JMIR Ment Health. (2015) 2:e5. 10.2196/mental.3649 26543911 PMC4607380

[47] Alonzo D Popescu M. Utilizing social media platforms to promote mental health awareness and help seeking in underserved communities during the COVID-19 pandemic. J Educ Health Promot. (2021) 10:156. 10.3103/jehp.jehp_21_21 34222531 PMC8224506

[48] Aikman K Brutt L de Ronde O Lim D Stratton P Wong M. Mass media campaigns for chronic pain: A scoping review to inform design of future campaigns. Physical Therapy Rev. (2020) 25:331–49. 10.1080/10833196.2020.1832711

[49] Rogers MA Lemmen K Kramer R Mann J Chopra V. Internet-delivered health interventions that work: systematic review of meta-analyses and evaluation of website availability. J Med Internet Res. (2017) 19:e90. 10.2196/jmir.7111 28341617 PMC5384996

[50] Ekwaru JP Ohinmaa A Dabravolskaj J Maximova K Veugelers PJ. Cost-effectiveness and return on investment of school-based health promotion programmes for chronic disease prevention. Eur J Public Health. (2021) 31:1183–9. 10.1093/eurpub/ckab130 34355754 PMC8643402

[51] Duijzer G Buckman A Groenveld A Jansen S Havenmen-Neis A Heinrich J. Cost-effectiveness of the SLIMMER diabetes prevention intervention in Dutch primary health care: economic evaluation from a randomised controlled trial. BMC Health Serv Res. (2019) 19:824. 10.1186/s12913-019-4529-8 31711499 PMC6849241

[52] Prezio EA Pagán JA Shuval K Culica D. The Community Diabetes Education (CoDE) program: cost-effectiveness and health outcomes. Am J Prev Med. (2014) 47:771–9. 10.1016/j.amepre.2014.08.016 25455119

[53] McRae I Esturas A Mihapoulos C Chieotles O Bucholc Chatterton ML. Cost-effectiveness of dementia prevention interventions. J Prev Alzheimers Dis. (2021) 8:210–7. 10.14283/jpad.2020.71 33569569

[54] Masters R Anwar E Collins B Cookson R Capewell S. Return on investment of public health interventions: a systematic review. J Epidemiol Community Health. (2017) 71:827–34. 10.1136/jech-2016-208141 28356325 PMC5537512

[55] Le LK. Cost-effectiveness evidence of mental health prevention and promotion interventions: a systematic review of economic evaluations. PLoS Med. (2021) 18:e1003606. 10.1371/journal.pmed.1003606 33974641 PMC8148329

[56] Egger G Binns A Rossner S. The emergence of “lifestyle medicine” as a structured approach for management of chronic disease. Med J Aust. (2009) 190:143–5. 10.5694/j.1326-5377.2009.tb02317.x 19203313

[57] Sarris J O'Neil A Coulson CE Schweitzer I Berk M. Lifestyle medicine for depression. BMC Psychiatry. (2014) 14:107. 10.1186/1471-244X-14-107 24721040 PMC3998225

[58] Qaseem A Wilt TJ McLean RM Forciea MA Physicians CGCotACo Denberg TD. Noninvasive treatments for acute, subacute, and chronic low back pain: a clinical practice guideline from the american college of physicians. Ann Intern Med. (2017) 166:514–30. 10.7326/M16-2367 28192789

[59] Afshin A Babalola D Mclean M Yu Z Ma W Chen C-Y. Information technology and lifestyle: a systematic evaluation of internet and mobile interventions for improving diet, physical activity, obesity, tobacco, alcohol use. J Am Heart Assoc. (2016) 5:9. 10.1161/JAHA.115.003058 27581172 PMC5079005

[60] Null G Pennesi L. Diet and lifestyle intervention on chronic moderate to severe depression and anxiety and other chronic conditions. Complement Ther Clin Pract. (2017) 29:189–93. 10.1016/j.ctcp.2017.09.007 29122259

[61] Altug Z. Lifestyle medicine for chronic lower back pain: an evidence-based approach. Am J Lifestyle Med. (2021) 15:425–33. 10.1177/1559827620971547 34366741 PMC8299916

[62] Nijs J D'Hondt E Clarys P Deliens T Polli A Malfliet A. Lifestyle and chronic pain across the lifespan: an inconvenient truth? PM R. (2020) 12:410–9. 10.1002/pmrj.12244 31437355

[63] Chehade L Jaafar ZA Masri DE Zmerly H Kreidieh D Tannir H. Lifestyle modification in rheumatoid arthritis: dietary and physical activity recommendations based on evidence. Curr Rheumatol Rev. (2019) 15:209–4. 10.2174/1573397115666190121135940 30666911

[64] Kivipelto Mangialasche MF Ngandu T. Lifestyle interventions to prevent cognitive impairment, dementia and Alzheimer disease. Nat Rev Neurol. (2018) 14:653–66. 10.1038/s41582-018-0070-3 30291317

[65] Mutie P Giordano G Franks P. Lifestyle precision medicine: the next generation in type 2 diabetes prevention? BMC Med. (2017) 15:171. 10.1186/s12916-017-0938-x 28934987 PMC5609030

[66] Kolb H Martin S., Environmental/lifestyle factors in the pathogenesis and prevention of type 2 diabetes. BMC Med. (2017) 15:131. 10.1186/s12916-017-0901-x 28720102 PMC5516328

[67] Frumkin H Bratman GN Breslow SJ Cochran B Jr PHK Lawler JJ. Nature contact and human health: a research agenda. Environ Health Perspect. (2017) 125:075001. 10.1289/EHP1663 28796634 PMC5744722

[68] Coventry PA Brown J Pervin J Barbyn S Palemen R Breedvelt J. Nature-based outdoor activities for mental and physical health: Systematic review and meta-analysis. SSM - Population Health. (2021) 16:100934. 10.1016/j.ssmph.2021.100934 34646931 PMC8498096

[69] Mcsweeney J Rainham D Johnson SA Sherry SB Singleton J. Indoor nature exposure (INE): a health-promotion framework. Health Promot Int. (2015) 30:126–39. 10.1093/heapro/dau081 25252597

[70] Hansen M Jones MR K Tocchini. Shinrin-Yoku (forest bathing) and nature therapy: a state-of-the-art review. Int J Environ Res Public Health. (2017) 14:851. 10.3390/ijerph14080851 28788101 PMC5580555

[71] Bratman GN Anderson CB Berman MG Cochran B de Vries S Flanders J. Nature and mental health: an ecosystem service perspective. Sci Adv. (2019) 5:eaax0903. 10.1126/sciadv.aax0903 31355340 PMC6656547

[72] Meredith GR Rakow DA Eldermire ERB Madsen CG Shelley SP Sachs NA. Minimum time dose in nature to positively impact the mental health of college-aged students, and how to measure it: a scoping review. Front Psychol. (2019) 10:2942. 10.3389/fpsyg.2019.02942 31993007 PMC6970969

[73] Kang B Kim T Kim MJ Lee KH Choi S Lee DH. Relief of chronic posterior neck pain depending on the type of forest therapy: comparison of the therapeutic effect of forest bathing alone versus forest bathing with exercise. Ann Rehabil Med. (2015) 39:957–63. 10.5535/arm.2015.39.6.957 26798610 PMC4720772

[74] Yau KK Loke AY. Effects of forest bathing on pre-hypertensive and hypertensive adults: a review of the literature. Environ Health Prev Med. (2020) 25:23. 10.1186/s12199-020-00856-7 32571202 PMC7310560

[75] Andersen L Corazon SSS Stigsdotter UKK. Nature exposure and its effects on immune system functioning: a systematic review. Int J Environ Res Public Health. (2021) 18:1416. 10.3390/ijerph18041416 33546397 PMC7913501

[76] Leubner D Hinterberger T. Reviewing the effectiveness of music interventions in treating depression. Front Psychol. (2017) 8:1109. 10.3389/fpsyg.2017.01109 28736539 PMC5500733

[77] Tang Q Huang Z Zhou H Ye P. Effects of music therapy on depression: A meta-analysis of randomized controlled trials. PLoS ONE. (2020) 15:e0240862. 10.1371/journal.pone.0240862 33206656 PMC7673528

[78] Garza-Villarreal EA Pando V Vuust P ParsonC. Music-induced analgesia in chronic pain conditions: a systematic review and meta-analysis. Pain Physician. (2017) 20:597–610. 10.36076/ppj/2017.7.597 29149141

[79] Lee JH. The effects of music on pain: a meta-analysis. J Music Ther. (2016) 53:430–77. 10.1093/jmt/thw012 27760797

[80] Gonzalez M Pascoe MC Yang G de Manincor M Grant S Lacey J. Yoga for depression and anxiety symptoms in people with cancer: A systematic review and meta-analysis. Psychooncology. (2021) 30:1196–208. 10.1002/pon.5671 33763925

[81] Zhu F. Yoga compared to non-exercise or physical therapy exercise on pain, disability, and quality of life for patients with chronic low back pain: A systematic review and meta-analysis of randomized controlled trials. PLoS One. (2020) 15:e0238544. 10.1371/journal.pone.0238544 32870936 PMC7462307

[82] Seshadri A. Exercise, yoga, and tai chi for treatment of major depressive disorder in outpatient settings: a systematic review and meta-analysis. Prim Care Companion CNS Disord. (2020) 23:20r02722. 10.4088/PCC.20r02722 33389843

[83] Zhu Y Wang R Tang X Li Q Xu G Zhang A. The effect of music, massage, yoga and exercise on antenatal depression: A meta-analysis. J Affect Disord. (2021) 292:592–602. 10.1016/j.jad.2021.05.122 34147972

[84] Denham-Jones L Gaskell L Spence N Pigott T. A systematic review of the effectiveness of yoga on pain, physical function, and quality of life in older adults with chronic musculoskeletal conditions. Musculoskeletal Care. (2021) 20:47–73. 10.1002/msc.1576 34125986

[85] Kim SD. Twelve weeks of yoga for chronic nonspecific lower back pain: a meta-analysis. Pain Manag Nurs. (2020) 21:536–42. 10.1016/j.pmn.2020.07.002 32830047

[86] Stier-Jarmer M Throner V Kirschneck M Immich G Frisch D Schuh A. The psychological and physical effects of forests on human health: a systematic review of systematic reviews and meta-analyses. Int J Environ Res Public Health. (2021). 18:(1770). 10.3390/ijerph18041770 33670337 PMC7918603

[87] Serrat M Almirall M Musté M Sanabria-Mazo JP Feliu-Soler A Méndez-Ulrich JL. Effectiveness of a multicomponent treatment for fibromyalgia based on pain neuroscience education, exercise therapy, psychological support, and nature exposure (NAT-FM): a pragmatic randomized controlled trial. J Clin Med. (2020) 9:3348. 10.3390/jcm9103348 33081069 PMC7603188

[88] Gong H Ni C Shen X Wu T Jiang C. Yoga for prenatal depression: a systematic review and meta-analysis. BMC Psychiatry. (2015) 15:14. 10.1186/s12888-015-0393-1 25652267 PMC4323231

[89] Long C Ye J Chen M Gao D Huang Q. Effectiveness of yoga therapy for migraine treatment: A meta-analysis of randomized controlled studies. Am J Emerg Med. (2022) 58:95–9. 10.1016/j.ajem.2022.04.050 35660369

[90] Kraak VI Kumanyika S Story M. The commercial marketing of healthy lifestyles to address the global child and adolescent obesity pandemic: prospects, pitfalls and priorities. Public Health Nutr. (2009) 12:2027–36. 10.1017/S1368980009990267 19545470

[91] Keygnaert I Dias S Stock C Frahsa A Dietrich K. Editorial: how can we co-create solutions in health promotion with users and stakeholders? Front Public Health. (2021) 9:773907. 10.3389/fpubh.2021.773907 34957026 PMC8692254

[92] Chaudhury P Banerjee D.“Recovering with nature”: a review of ecotherapy and implications for the COVID-19 pandemic. Front Public Health. (2020) 8:604440. 10.3389/fpubh.2020.604440 33363096 PMC7758313

[93] Hinde S Bojke L Coventry P. The cost effectiveness of ecotherapy as a healthcare intervention, separating the wood from the trees. Int J Environ Res Public Health. (2021). 18:11599. 10.3390/ijerph182111599 34770112 PMC8582680

[94] Maller C Townsend M Pryor A Brown P Leger LS. Healthy nature healthy people: ‘contact with nature' as an upstream health promotion intervention for populations. Health Promotion Int. (2005) 21:45-54. 10.1093/heapro/dai032 16373379

[95] Gallagher KM Updegraff JA. Health message framing effects on attitudes, intentions, and behavior: a meta-analytic review. Ann Behav Med. (2012) 43:101–16. 10.1007/s12160-011-9308-7 21993844

[96] Ainiwaer A Zhang S Ainiwaer X Ma F. Detection behaviors and attitudes: systematic review and meta-analysis. J Med Internet Res. (2021) 23:e27634. 10.2196/27634 34528887 PMC8485193

[97] Schriewer K Bulaj G. Music streaming services as adjunct therapies for depression, anxiety, and bipolar symptoms: convergence of digital technologies, mobile apps, emotions, and global mental health. Front Public Health. (2016) 4:217. 10.3389/fpubh.2016.00217 27747209 PMC5043262

[98] Gadd S Tak C Bulaj G. Developing music streaming as an adjunct digital therapy for depression: A survey study to assess support from key stakeholders. J Affect Disord. (2020) 2:100048. 10.1016/j.jadr.2020.100048

[99] Afra P Bruggers CS Sweney M Fagatele L Alavi F Greenwald M. Mobile software as a medical device (SaMD) for the treatment of epilepsy: development of digital therapeutics comprising behavioral and music-based interventions for neurological disorders. Front Hum Neurosci. (2018) 12:171. 10.3389/fnhum.2018.00171 29780310 PMC5946004

[100] Chai PR Carreiro S Ranney ML Karanam K Ahtisaari M Edwards R. Music as an adjunct to opioid-based analgesia. J Med Toxicol. (2017) 13:249–254. 10.1007/s13181-017-0621-9 28646359 PMC5570730

[101] Sesso G Sicca F Safe and sound: meta-analyzing the Mozart effect on epilepsy. Clin Neurophysiol. (2020) 131:1610–20. 10.1016/j.clinph.2020.03.039 32449680

[102] Dascal J Reid M IsHak WW Spiegel B Recacho J Rosen B. Virtual reality and medical inpatients: a systematic review of randomized, controlled trials. Innov Clin Neurosci. (2017) 14:14–21. 28386517 PMC5373791

[103] Schwartz LM Woloshin S. Medical marketing in the United States, 1997-2016. JAMA. (2019) 321:80–96. 10.1001/jama.2018.19320 30620375

[104] Tal A Wansink B. An apple a day brings more apples your way: healthy samples prime healthier choices. Psychol Marketing. (2015) 32:575–84. 10.1002/mar.20801

[105] Feteira-Santos R Fernandes J Virgolino A Alarcão V Sena C Vieira CP. Effectiveness of interpretive front-of-pack nutritional labelling schemes on the promotion of healthier food choices: a systematic review. Int J Evid Based Healthc. (2020) 18:24–37. 10.1097/XEB.0000000000000214 31895716

[106] Huntsman DD Bulaj G. Healthy Dwelling: design of biophilic interior environments fostering self-care practices for people living with migraines, chronic pain, and depression. Int J Environ Res Public Health. (2022) 19:2248. 10.3390/ijerph19042248 35206441 PMC8871637

[107] Desai V. Digital marketing: a review. Int J Trend Res Dev. (2019) 5:196–200. 10.31142/ijtsrd23100

[108] Moin S. Brand storytelling: a review of the interdisciplinary literature. Brand Storytelling in the Digital Age. (2020) 2020:19–39. 10.1007/978-3-030-59085-7_2

[109] Petrovic M Banonno S M Ionio C Hagedoorn M Gaggioli A. Using the transformative storytelling technique to generate empowering narratives for informal caregivers: semistructured interviews and method demonstration. JMIR Form Res. (2022) 6:e36405. 10.2196/36405 35802492 PMC9382549

[110] Lohr AM Tapia JPR Valdez ES Hassett LC Gubrium AC Fiddian-Green A. The use of digital stories as a health promotion intervention: a scoping review. BMC Public Health. (2022) 22:1180. 10.1186/s12889-022-13595-x 35698097 PMC9192132

[111] Mills JM Guy JW Oestreich JH. Digital storytelling review in a pharmacy self-care course. Pharmacy (Basel). (2022) 10:45. 10.3390/pharmacy10020045 35448704 PMC9032475

[112] Rossi J Yudell M. The use of persuasion in public health communication: an ethical critique. Public Health Ethics. (2012) 5:192–205. 10.1093/phe/phs019

[113] Lin CY. Promote health or prevent disease? The effects of health-related advertising on eating behavior intention. Int J Environ Res Public Health. (2015) 12:3517–34. 10.3390/ijerph120403517 25826394 PMC4410200

[114] Gleeson D Menkes DB. Trade agreements and direct-to-consumer advertising of pharmaceuticals. Int J Health Policy Manag. (2018) 7:98–100. 10.15171/ijhpm.2017.124 29524933 PMC5819384

[115] Biegler P Vargas P. Feeling is believing: evaluative conditioning and the ethics of pharmaceutical advertising. J Bioeth Inq. (2016) 13:271–9. 10.1007/s11673-016-9702-8 26818244

[116] Gilbody S Wilson P Watt I. Benefits and harms of direct to consumer advertising: a systematic review. Qual Saf Health Care. (2005) 14:246–50. 10.1136/qshc.2004.012781 16076787 PMC1744049

[117] Zachry WM Ginsburg DB. Patient autonomy and the regulation of direct-to-consumer advertising. Clin Ther. (2001) 23:2024–37. 10.1016/S0149-2918(01)80155-7 11813936

[118] Bharadwaj. Regulatory exposure of deceptive marketing and its impact on firm value. J Mark. (2009) 73:227–43. 10.1509/jmkg.73.6.227

[119] Bladow LE. Worth the click: why greater FTC enforcement is needed to curtail deceptive practices in influencer marketing. Wm. & *Mary L. Rev*. (2017) 59:1123.

[120] Clar C Dyakova M Curtis K Dawson C Donnelly P Knifton L. Just telling and selling: current limitations in the use of digital media in public health: a scoping review. Public Health. (2014) 128:1066–75. 10.1016/j.puhe.2014.09.009 25443388

